# Revisiting the psychometric properties of the Scoliosis Research Society-22 (SRS-22) French version

**DOI:** 10.1186/s13013-017-0129-8

**Published:** 2017-07-17

**Authors:** Jean Théroux, Norman Stomski, Stanley Innes, Ariane Ballard, Christelle Khadra, Hubert Labelle, Sylvie Le May

**Affiliations:** 1 Research Center, Sainte-Justine University Hospital Center, Montreal, QC Canada; 2 School of Health Profession, Murdoch University, 90, South Street, Murdoch, WA 6150 Australia; 3 Faculty of Nursing, University of Montreal, Montreal, QC Canada; 4 Faculty of Medicine, University of Montreal, Montreal, Canada

**Keywords:** SRS-22, Adolescent idiopathic scoliosis, Reliability, Validity, Psychometrics

## Abstract

**Background:**

Adolescent idiopathic scoliosis (AIS) is among the most common spinal deformities affecting adolescents. The Scoliosis Research Society-22 questionnaire is commonly used to assess health-related quality of life in AIS patients, including pain.

The objective of this study is to verify the psychometric properties of the Scoliosis Research Society-22 French version (SRS-22fv) questionnaire.

**Methods:**

A prospective methodological design was used to verify the psychometric properties of the French version of the SRS-22fv. Participants were initially recruited from the orthopaedic scoliosis department at Sainte-Justine Hospital (Montreal, Canada) and completed the SRS-22fv and the SF-12 questionnaire. The SRS-22fv’s structure was evaluated through principal component analysis (PCA). Linear regression was used to assess convergent validity between the SRS-22fv and the SF-12.

**Results:**

Data was available from 352 participants with AIS. Most participants were female (87%, *n* = 307), and the average age was 14.3 (SD = 1.8) years. The mean thoracic and lumbar Cobb angles were 27.9° (SD = 3.3) and 23.6° (SD = 9.4), respectively. Overall, 71.4% (*n* = 252) of the participants presented with spinal pain. About one-third (29%) reported thoracic pain, and almost half (44%) experienced lumbar pain. The PCA identified four redundant items, which resulted in a modified 18-item questionnaire. In comparison to the original questionnaire, the modified version showed higher levels of internal consistency for four of the five factors, explained a greater proportion of the total variance (63.3%), and generated higher inter-item total correlations.

**Conclusion:**

We propose a shorter version of the SRS-22fv, thus the Canadian SRS-18fv, which showed an improved internal consistency and scale structure compared to the original SRS-22fv. We believe that this modified version would be better suited to assess the quality of life of adolescents with idiopathic scoliosis.

## Background

Adolescent idiopathic scoliosis (AIS) is among the most common spinal deformities affecting children and adolescents [1]. This deformity is defined as a three-dimensional structural lateral deviation of the spine greater than 10° with an associated prevalence of 2–3% in adolescents [2].

Initially developed by Haher et al. [3] and the Scoliosis Research Society (SRS), the initial SRS-24 questionnaire was designed to evaluate disease-specific health-related quality of life (HRQoL) in AIS patients who were scheduled to undergo spinal fusion surgery. The original version of this questionnaire contained 24 items with an underlying structure of six different factors (pain, general self-image, postoperative self-image, postoperative function, function from back condition, general level of activity, and satisfaction). The SRS-24 underwent further refinement [4] and was modified to a 23-item questionnaire with five factors instead of six.

To obtain a more reliable disease-specific instrument, the SRS questionnaire was further modified to a 22-item version [5, 6]. The final version of this questionnaire (SRS-22r) was revised in 2006. [7] The SRS-22r includes 22 items distributed among five different factors (pain, self-image/appearance, function/activity, mental health, and satisfaction with management). Each component includes five items, except for the “satisfaction with management”, which only contains two items.

The SRS-22 has become one of the most widely used instruments in assessing the quality of care for individuals with scoliosis and has been validated in adolescent and adult populations [8, 9]. It has been translated and adapted in multiple languages over the last decade [10–26], among which a French-Canadian version (SRS-22fv) was developed and validated by Beauséjour et al. [18] in 2009. Several issues arise from that French validation study, particularly cross-loading of numerous items and items loading in factors that differed from the original English version. Hence, we consider that it would be important to reassess the psychometric properties of the SRS-22fv. The aim of this study was to verify the structure, reliability, and construct validity of the SRS-22fv.

## Methods

The present study is part of a larger prospective study examining back pain [27]. Overall, 500 consecutive participants were enrolled in the study, but the completed SRS-22fv questionnaire data were only obtained from 352 participants, which met the requirement for factor analysis. The study took place at Sainte-Justine University Hospital Centre (Montreal, Canada) which is the largest mother and child centre in Canada and received approval by the Institutional Research Ethics Board of Sainte-Justine’s Research Centre. Questionnaires were completed by patients on their first or subsequent visit at the scoliosis orthopaedic clinic from October 2014 until May 2015. Patients were included if they were aged between 10 and 17 years and had received a diagnosis of adolescent idiopathic scoliosis with a spinal curve (Cobb angle) of at least 10°. Patients were excluded if they had (1) a spinal deformity other than AIS, (2) a congenital or acquired spinal abnormality, (3) previous spinal surgery, (4) a diagnose of mental disability, or (5) sustained a significant spinal trauma within the last 12 months.

### Analyses

All analyses were performed with SPSS v22 (IBM, Armonk, NY, USA). According to instrument validation guidelines [28], 10 participants per item are needed to undertake a robust factor analysis. Considering that the SRS-22fv has 22 items, a minimum sample size of 220 participants was required.

The adapted French-Canadian SRS-22fv was summed and scored in agreement with the original English version on a five-point Likert scale (5 = best; 1 = worst). As such, the minimum and maximum score range is from 5 to 25, except for the “satisfaction with management” domain which contains only two items. Mean scores, internal consistency, and ceiling and floor effects were calculated for each factor. Construct validity was assessed with multiple regression comparing corresponding factors of the SRS-22fv and SF-12 [29, 30].

Principal component analysis [31] with a promax rotation was used to explore the structure of SRS-22fv. Items with loadings below 0.45, cross-loadings above 0.32, or inter-item correlations below 0.30 were individually deleted, and the component solution was then re-extracted [32]. Internal consistency was examined by calculating Cronbach’s alpha for each factor and the total questionnaire score.

## Results

### Characteristics of participants

More than 70% (352/500) of the participants returned a completed questionnaire and thus were eligible for analysis. Participants’ characteristics are described in Table 1. Most participants were female (87%) (*n* = 307), with a mean age of 14.3 (SD = 1.8) years, and had a mean thoracic and lumbar Cobb angles of 26.6° (SD = 12.8) and 23.6° (SD = 9.4), respectively. About one-third (31%, *n* = 109) of the participants attended the orthopaedic clinic for the first time, and almost half (40%, *n* = 142) were using braces.

**Table 1:** Characteristics of participants

	All (*n* = 352)	Girls (*n* = 307)	Boys (*n* = 45)
Age (mean) (SD)	14.26 (1.76)	14.25 (1.73)	14.33 (1.93)
Brace (yes) *n* (%)	142 (40)	125 (41)	17 (38)
First visit (yes) (%)	109 (31)	92 (30)	17 (38)
Mean Cobb angle (SD) in degrees			
Thoracic principal	27.87° (13.30)	27.93° (13.37)	26.64° (12.81)
Thoraco-lumbar	23.93° (11.14)	24.38° (11.19)	21° (10.90)
Lumbar	23.61° (9.40)	23.72° (9.15)	22.88° (11.20)
Angle of trunk rotation in degrees			
Dorsal	8.52° (4.16)	8.40° (4.15)	9.60° (4.18)
Lumbar	7.2° (4)	7° (3.81)	8.60° (5.4)

There were no statistical differences on all the variables between genders

### Mean score of SRS-22fv factors

Mean scores for each of the factors of the SRS-22fv are presented in Table 2. The total mean SRS-22fv score (4.14 [SD = 0.45]) indicated that the participants, in general, had a high level of well-being. No floor effects were observed but moderate ceiling effects were noted for two factors (satisfaction with management and self-image).

**Table 2:** Mean scores among all the factors of the SRS-22fv (*n* = 352)

	Pain	Self-image	Function	Mental health	Satisfaction with management	Total (1–22)
Mean score (SD)	4.32 (0.65)	3.93 (0.62)	4.32 (0.41)	4.14 (0.66)	3.75 (0.89)	4.14 (0.45)
SEM	0.034	0.033	0.021	0.035	0.048	0.02
% floor	0	0	0	0	1.7	
% ceiling	22.8	5.4	0.9	11.1	17.1	

*SEM* standard error of the mean

### Psychometric properties of the SRS-22fv

The psychometric properties of the SRS-22fv were considered through examining the loading of each item on discrete factors, item cross-loading between factors, inter-item correlations, item floor and ceiling effect, and communality values. The initial principal component analysis (PCA) (Table 3) revealed that item 15 had an inter-item correlation below 0.30. That item was deleted, and the factors were re-extracted, which showed that item 12 cross-loaded and that item 18 did not load on any component. Item 18 was deleted, and PCA was performed once again, which demonstrated that item 12 cross-loaded. After removing item 12 and re-extracting the factor solution, it was found that item 19 was redundant since its inter-item correlation fell below 0.30. The next iteration of the PCA revealed that all remaining items strongly loaded on discrete factors and all inter-item correlations exceeded 0.30. This resulted in a revised 18-item questionnaire (Table 4) that explained more of the variance than the original SRS-22fv item questionnaire (63.3% compared to 55.7%). In addition, four of the scales, derived from the final factor solution, demonstrated higher levels of internal consistency than the corresponding scales in the SRS-22fv (Table 5). Finally, for the revised 18-item questionnaire, the Kaiser-Meyer-Olkin value (0.87) was well above the minimum criterion of 0.5, and the Bartlett test of sphericity was statistically significant (*p* = 0.000).

**Table 3:** Factor structure of the SRS-22fv

	1	2	3	4	5
Q3: During the past 6 months, have you been a very nervous person?^a^	*.757*	.168	−.063	−.082	−.076
Q7: In the past 6 months, have you felt so down in the dumps that nothing could cheer you up?	*.682*	.007	.034	.055	−.046
Q13: Have you felt calm and peaceful during the last six months?	*.753*	.087	−.114	−.034	.095
Q16: In the past six months, have you felt down hearted and blue?	*.845*	−.026	.070	.008	.052
Q20: Have you been a happy person during the past six months?	*.699*	−.030	.174	−.100	.026
Q1: Which of the following best describes the amount of pain you have experienced during the past 6 months?	.204	*.766*	−.147	.102	−.038
Q2: Which one of the following best describes the amount of pain you have experienced over the last month?	.179	*.807*	−.165	.126	−.001
Q8: Do you experience back pain when at rest?	.050	*.717*	.016	−.103	−.127
Q11: Which one of the following best describes your medication usage for your back?	−.174	*.479*	.076	.196	.096
Q17: In the past three months, have you taken any sick days from work/school due to back pain and, if so, how many?	−.166	*.333*	.001	.267	(.458)
Q4: If you had to spend the rest of your life with your back as it is right now, how would you feel about it?	−.002	(.486)	*.367*	.029	.008
Q6: How do you look in clothes?	.152	−.110	*.673*	.183	−.029
Q10: Which of the following best describes the appearance of your trunk, defined as the human body except for the head and extremities?	.021	.088	*.730*	.055	.000
Q14: Do you feel that your condition affects your personal relationships?	.275	−.093	*.383*	.196	.176
Q19: Do you feel attractive with your current back condition?	−.047	−.172	*.441*	.095	.181
Q5: What is your current level of activity?	−.031	−.052	.060	*.766*	−.105
Q9: What is your current level of work/school activity?	−.076	.130	.051	*.759*	.009
Q12: Does your back limit your ability to do things around the house?	.036	(.358)	−.003	*.539*	.038
Q15: Are you and/or your family experiencing financial difficulties because of your back?	.112	−.230	.117	*−.023*	(.777)
Q18: Does your back condition limit your going out with friends/family?	.029	−.165	.078	*.544*	(−.585)
Q21: Are you satisfied with the results of your back management?	−.117	(.422)	(.583)	−.198	*−.102*
Q22: Would you have the same management again if you had the same condition?	−.066	(.480)	(.484)	−.181	*−.018*

Italicized data indicates items of the original questionnaire

Items in parenthesis () represent items loading on the inappropriate factor as compared to the original English version

^a^Questions from the original English version

**Table 4:** Factor structure of the SRS-18fv

	1	2	3	4	5
	MH	Pain	SI	Func	Satis
Q3	.726				
Q7	.749				
Q13	.764				
Q16	.837				
Q20	.584				
Q1		.877			
Q2		.863			
Q4		.439			
Q8		.795			
Q11		.476			
Q6			.849		
Q10			.779		
Q14			.719		
Q5				.749	
Q9				.795	
Q17				.599	
Q21					.846
Q22					.850

% explained variance 63.30%

Residual ≥0.05, 31%; determinant = 0.0001

*MH* mental health, *SI* self-image, *Func* function, *Satis* satisfaction with management

**Table 5:** Internal consistency comparison

Factor		Current study	
	Beauséjour [18]	SRS-22fv	SRS-18fv
Pain	0.79	0.76	0.80
Self-image	0.67	0.66	0.74
Function	0.68	0.60	0.61
Mental health	0.79	0.83	0.83
Satisfaction with management	0.69	0.71	0.71

Table 6 displays the results of linear regression analysis undertaken to examine the convergent validity of the revised 18-item SRS. Results showed that the scales in the revised questionnaire were significantly associated with conceptually similar scales in the SF-12. These findings provide additional support to the factor structure of the revised questionnaire.

**Table 6:** Construct validity of the SRS-18fv with corresponding SF-12 factors

	SRS-18fv (*β*; 95% CI)				
SF-12	Pain	Self-image	Function	Mental health	Satisfaction with management
Physical functioning	0.67; 0.19–1.15	0.59; 0.30–0.88			
Role functioning	0.70; 0.31–1.08	0.54; 0.31–0.77			
Bodily pain	1.00; 0.56–1.43	0.36; 0.10–0.62			0.45; 0.17–0.73
General health perception		0.39; 0.17–0.62	−0.30;−0.49 to −0.11		
Vitality		0.64; 0.31–0.96			
Social functioning		0.62; 0.33–0.92		0.59; 0.14–1.04	
Role emotional		0.52; 0.29–0.75			
Mental health		0.47; 0.25–0.69	−0.28;−0.46 to −0.09	0.68; 0.34–1.02	

Results not reported were not significant

## Discussion

This study reexamined the psychometric properties of the adapted French-Canadian version of the SRS-22. Our results identified four redundant items in the SRS-22fv, which after deletion improved the total variance explained by the five factors, while also enhancing the internal consistency of four of the five factors. Hence, our proposed SRS-18fv is more parsimonious and psychometrically robust than the SRS-22fv.

Adolescent idiopathic scoliosis evaluation is no longer viewed solely based on treatment procedures (observation, bracing, or surgery), but also requires assessment of health-related quality of life [7, 33]. The original SRS-22 questionnaire is currently widely utilised to assess HRQoL in adolescents with scoliosis, [10–26, 30, 34–41] but the findings of the present study indicate that our revised SRS-18fv may be more appropriate for the French-Canadian adolescent population.

Our findings were consistent with the previous study by Beauséjour et al. [18] that validated the SRS-22 in a French-Canadian population. Both our results and Beauséjour’s [18] identified several cross-loading items and several items that loaded on factors that differed from the English version of the SRS-22. But Beauséjour et al. [18] did not delete poorly fitted items and re-extract the factors, which is inconsistent with psychometric guidelines [32]. The authors also noted that the SRS-22 was suitable to use without any revisions. In our view, however, the identification of poorly performing items in the present study and the previous study underlines the need to use a revised questionnaire that defines health-related quality of life constructs of importance to adolescents with scoliosis in a clearly delineated manner.

Regarding the internal consistency, only one factor, “function”, in the SRS-18fv had a Cronbach alpha value below 0.70, the minimally acceptable level [42]. Other studies of the original SRS-22 and SRS-22fv also reported Cronbach alpha values for the function factor that fell below the accepted value. [12, 26, 30, 35, 39] This suboptimal level of internal consistency suggests that items within this component may not all be clearly tapping the same construct. The lack of clear conceptual delineation might arise from the inclusion of terms like “labor” and “work” in the phrasing of the items. This wording may not be readily comprehended or deemed relevant by children and adolescents, particularly considering that one-third of our sample was aged 13 years or below.

Notably, items 4 and 17 of the SRS-18fv loaded on different factors than in the English version of the SRS-22, which may be influenced by differences in interpretation of the items following translation from English to French. Item 4: “If you had to spend the rest of your life with your back shape as it is right now, how would you feel about it?” originally associated with “self-image” loaded on the “pain” component. This inconsistency could be related to the meaning attributed to the term “back shape” in French. In the translated French version, the adolescents’ interpretation of this item might be more closely associated with “pain” than the actual “shape” of their back. Hence, item 4 in the French translation could be revised to be more congruent with the initial English meaning. Item 17: “In the last 3 months have you taken any days off of work, including household work, or school because of back pain” originally associated with “pain” loaded on the “function” component. This divergence in loading may result from the interpretation that pain and function are conceptually similar since they are related to physical limitations. These interpretation issues suggest that it might be worthwhile to reexamine the content validity of the initial French version of the SRS-22.

In the previous validation study of the SRS-22fv [18], construct validity was examined by performing Pearson correlations between the related SRS-22 and the SF-12 domains. However, the use of such correlations does not control for covariance and also increase the risk of type II errors [43]. In the present study, we established construct validity using linear regression, which controls for covariance and adjusts for multiple comparisons which result in a more robust assessment.

### Study limitations

Several caveats should be considered in interpreting this study’s findings. First, this study enrolled participants from a single institution, which may impact on the generalisability of the results. However, the institution from which participants were recruited is the most important mother and child centre in Canada and therefore receives a diversified adolescent population from many different regions of this province. Second, participants were all pre-surgical cases with a Cobb angle inferior to 45°. Consequently, results should be interpreted with caution in a population with more severe Cobb angles. Third, the test-retest reliability of our revised questionnaire warrants examination in further studies. Finally, the construct validity of our revised questionnaire was assessed with the SF-12 questionnaire which has yet to be validated in a French adolescent population.

## Conclusion

Our revised questionnaire is briefer and more psychometrically robust than the original version of the SRS-22fv. It provides clinicians and researchers with a better tool to understand the impact of scoliosis on adolescents’ health-related quality of life.

## Abbreviations

AIS: Adolescent idiopathic scoliosis
HRQoL: Health-related quality of life
PCA: Principal component analysis
SD: Standard deviation
SRS: Scoliosis Research Society
